# Future temperature and salinity do not exert selection pressure on cyst germination of a toxic phytoplankton species

**DOI:** 10.1002/ece3.5009

**Published:** 2019-04-01

**Authors:** Jacqueline Jerney, Sanna Suikkanen, Elin Lindehoff, Anke Kremp

**Affiliations:** ^1^ Marine Research Centre Finnish Environment Institute Helsinki Finland; ^2^ Tvärminne Zoological Station University of Helsinki Hanko Finland; ^3^ Department of Biology and Environmental Science, Linnaeus University Centre of Ecology and Evolution in Microbial Model Systems, EEMiS Linnaeus University Kalmar Sweden; ^4^ Leibniz‐Institut für Ostseeforschung Warnemünde Rostock Germany

**Keywords:** adaptation, *Alexandrium ostenfeldii*, climate change, dinoflagellates, excystment, resting stage

## Abstract

Environmental conditions regulate the germination of phytoplankton resting stages. While some factors lead to synchronous germination, others stimulate germination of only a small fraction of the resting stages. This suggests that habitat filters may act on the germination level and thus affect selection of blooming strains. Benthic “seed banks” of the toxic dinoflagellate *Alexandrium ostenfeldii* from the Baltic Sea are genetically and phenotypically diverse, indicating a high potential for adaptation by selection on standing genetic variation. Here, we experimentally tested the role of climate‐related salinity and temperature as selection filters during germination and subsequent establishment of *A. ostenfeldii* strains. A representative resting cyst population was isolated from sediment samples, and germination and reciprocal transplantation experiments were carried out, including four treatments: Average present day germination conditions and three potential future conditions: high temperature, low salinity, and high temperature in combination with low salinity. We found that the final germination success of *A. ostenfeldii* resting cysts was unaffected by temperature and salinity in the range tested. A high germination success of more than 80% in all treatments indicates that strains are not selected by temperature and salinity during germination, but selection becomes more important shortly after germination, in the vegetative stage of the life cycle. Moreover, strains were not adapted to germination conditions. Instead, highly plastic responses occurred after transplantation and significantly higher growth rates were observed at higher temperature. High variability of strain‐specific responses has probably masked the overall effect of the treatments, highlighting the importance of testing the effect of environmental factors on many strains. It is likely that *A. ostenfeldii* populations can persist in the future, because suitable strains, which are able to germinate and grow well at potential future climate conditions, are part of the highly diverse cyst population.

**OPEN RESEARCH BADGES:**

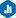

This article has earned an Open Data Badge for making publicly available the digitally‐shareable data necessary to reproduce the reported results. The data is available at https://doi.org/10.5061/dryad.c8c83nr.

## 1 INTRODUCTION

In temporally variable environments, the formation of dormant resting stages (propagules) is an effective strategy to secure survival of individual organisms (Lennon & Jones, 2011; Marcus, 1998) and protect populations against short‐ and long‐term environmental fluctuations (Ellegaard & Ribeiro, 2018; Morris et al., 2008). Dormant propagule banks integrate genetically and physiologically varying individuals produced in different seasons and years and form genetic reservoirs that exceed the diversity of active populations (Brendonck & De Meester, 2003). Propagule banks are thus an important factor defining the potential of organisms and populations to persist through environmental change.

Many phytoplankton species form dormant resting stages to survive unfavorable conditions (Fryxell, 1983). Dinoflagellates, a group of phytoplankton that can cause harmful algal blooms, produce fossilizable resting cysts (Dale, 1983). These can survive in sediments of lakes and oceans for many years and even decades (Ribeiro et al., 2011). Their germination may be endogenously controlled (Fischer et al., 2018), but typically defined environmental settings, often representative of suitable growth conditions for the respective species (Rengefors & Anderson, 1998) trigger excystment. Germination may be highly synchronized, leading to excystment of the entire viable seed pool once the right trigger is present (Anderson & Rengefors, 2006; Genovesi et al., 2009). Alternatively, only fractions of the cyst pool may germinate at specific conditions along an environmental gradient (Anglès, Garcés, Reñé, & Sampedro, 2012; Kim, Park, & Han, 2002; Moore et al., 2015) suggesting that cysts with diverse germination requirements are present in the sediment. At the same time, high genetic and phenotypic diversity has been observed in a dinoflagellate cyst pool from Baltic Sea sediments (Kremp et al., 2016).

Selection from standing genetic variation is an important mechanism of evolutionary adaptation whereby a population develops toward a phenotype that best fits the present environment (Orr, 2005). The ability to adapt is an important prerequisite to persist under stressful conditions or realize ecological opportunities arising from climate change (Hoffmann & Sgrò, 2011). The co‐occurrence of different phenotypic or adaptive traits in a population allows for natural selection of the best suitable genotype under certain environmental conditions. Studies on plants provide evidence that environmental factors exert natural selection on germination (Donohue et al., 2010). Selection for increased fecundity, for example, favors early germination, and selection on mortality can favor either early or delayed germination, depending on when a mortality event occurs (Donohue et al., 2010). The environment can thus act as a filter, which only allows individuals with a particular trait or phenotype to establish and persist, excluding all others (Kraft et al., 2015). Such environmental filters were shown to prevent germination of plant seeds in the season of highest threat for seedling establishment (Fernández‐Pascual et al., 2017). It is likely that also the trait‐dependent release of phytoplankton into the water column is regulated by respective environmental filters that prevent germination of genotypes which cannot adjust to unsuitable growth conditions.

Here, we investigate this hypothesis using the dinoflagellate *Alexandrium ostenfeldii *(Paulsen) Balech and Tangen, which forms toxic blooms in shallow coastal waters of the Baltic Sea, as a model species. Like many dinoflagellates from seasonal environments, *A. ostenfeldii* produces resting cysts as part of its life cycle and the cyst pool in coastal sediments was found to be genetically diverse (Tahvanainen et al., 2012). At the same time, considerable variation in temperature and salinity‐related traits occurred among individuals recruited from respective cysts (Kremp et al., 2016; Suikkanen, et al., 2013) which was suggested to serve adaptation of *A. ostenfeldii *populations to changing climate conditions in the Baltic Sea (Kremp et al., 2016).

The Baltic Sea is one of the largest brackish water bodies in the world, with a salinity gradient ranging from around 30 to 1 (BACC, 2008; Meier et al., 2006). Due to global warming an overall reduction of sea surface salinity by 1 to 3 psu, depending on the geographic region, is expected in the future (Meier et al., 2012, 2006). Besides salinity, temperature is a key environmental factor and for the total basin of the Baltic Sea a mean annual temperature increase of 3 to 5°C has been suggested for the late 21st century (Graham et al., 2008). This increase might be even more pronounced in stratified shallow coastal embayments of the Baltic Sea, which are a preferred habitat of *A. ostenfeldii*. Increasing summer temperatures may aid expansion of this species in the future (Kremp et al., 2012), which could be problematic due to its ability to produce several potent toxins (Martens et al., 2016; Salgado et al., 2015; Van de Waal et al., 2015), with demonstrated effects, like rapid behavioral disturbance and incapacitation, on co‐occurring biota (Sopanen et al., 2011). A thorough understanding of the mechanisms regulating the response of the species to the predicted changes is crucial to forecast its impact under future climate conditions.

In this study, we experimentally tested the role of climate‐related salinity and temperature changes as selection filters during germination and subsequent establishment of *A. ostenfeldii* strains. We were aiming to define if certain strains from the diverse cyst pool are selected by future temperature and salinity conditions and if selection acts at the level of cyst germination or later in the growth phase of the life cycle. Additionally, we investigated if strains which germinated at a specific temperature or salinity are adapted to those conditions or if their phenotypic response depends on the conditions after germination. We hypothesized that (a) temperature and salinity affect the final germination success (i.e., the maximum germination success at a given condition after four weeks) of *A. ostenfeldii* resting cysts and (b) successfully germinated strains are adapted to respective temperature and salinity conditions and will grow best at the same conditions. To test our hypotheses, germination experiments were carried out with wild resting cysts, isolated from sediments in a well‐described northern Baltic bloom site, and subsequent growth of resulting strains was recorded. Reciprocal transplantation experiments were carried out with successfully germinated strains to assess if *A. ostenfeldii* genotypes are adapted to germination conditions and therefore selected, or if they can acclimate to new conditions rapidly.

## 2 MATERIAL AND METHODS

We have strong support from amplified fragment length polymorphism and microsatellite data, showing that 95%–100% of Baltic Sea *A. ostenfeldii* strains established from a single resting cyst are unique (Tahvanainen et al., 2012, J. Jerney et al., unpublished), to assume that strains represent individual genotypes. Therefore, the terms “strain” and “genotype” are used synonymously in this study.

### 2.1 Sampling

Sediment samples were collected in September 2015 from a shallow embayment in the Föglö archipelago, Åland, Northern Baltic Sea (60°05, 6′N, 20°32, 4′E) which has been described in detail earlier (Hakanen et al., 2012; Kremp et al., 2009) and corresponds to station 4 in the latter reference. Sediment cores were taken from the very shallow bay (water depth <3 m) with a gravity corer (Limnos, Turku), and the uppermost flocculent sediment layer was transferred to 50 ml centrifugation tubes (Falcon). The tubes were entirely filled up with sediment and stored at cold (4°C) and dark conditions until further use to prevent germination of resting cysts before starting the experiments.

### 2.2 Sediment processing

**Figure 1:**
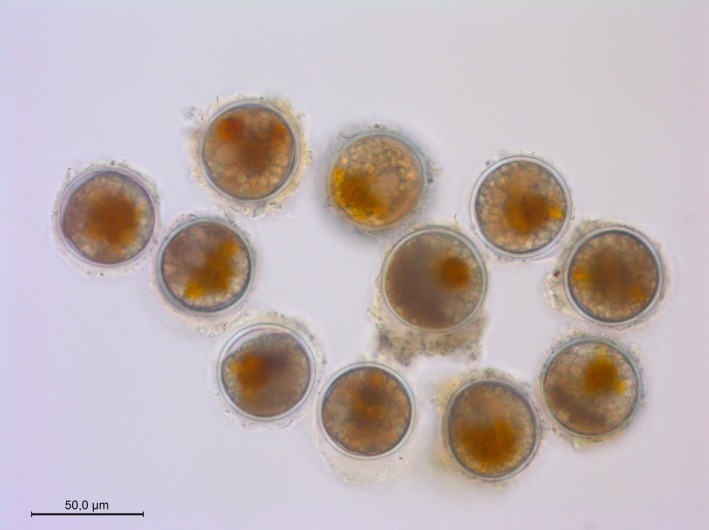
Typical resting cysts of the toxin producing marine dinoflagellate *Alexandrium ostenfeldii*, isolated from sediment sampled at the Åland Islands in the Baltic Sea

To detach resting cysts from sediment particles, subsamples were sonicated prior to the experiments for 30 s on constant duty cycle with a frequency of 20 kHz (Brandelin Sonoplus sonicator HD 2200). During sonication, the samples were cooled with ice to avoid temperature increase and sieved afterward to isolate the 30–76 µm fraction, containing *A. ostenfeldii* resting cysts. The material retained on the 30 µm screen was transferred into a 15 ml polypropylene centrifuge tube (Falcon) and diluted with filtered seawater (6 psu) to obtain a cyst slurry for microscopic isolation of single cysts (Figure 1).

### 2.3 Germination success

To determine if temperature or salinity affect the germination rate, a first germination experiment was carried out in March 2016. For this purpose, subsamples of the cyst slurry were inspected microscopically (Leica DMI3000 B inverted microscope) to obtain *A. ostenfeldii* cysts. Single resting cysts were isolated with a glass micropipette and transferred to 24‐well plates, each well filled with 2 ml of f/2‐Si culture medium (Guillard, 1975; Guillard & Ryther, 1962) prepared from filtered (0.2 µm), autoclaved Baltic Sea water. For each treatment, three replicates, with 10 cysts each, were used. Isolated cysts were incubated at the following conditions: Control (C) 16°C, 6 psu, (representing average germination conditions in the Baltic Sea); high temperature (T) 20°C, 6 psu; low salinity (S) 16°C, 3 psu; and high temperature combined with low salinity (TS) 20°C, 3 psu. Cysts were incubated at 14:10 hr light:dark cycle; and a light intensity of ~100 µmol photons m^−2^ s^−1^. Germination success was recorded every 2–3 days microscopically, during a period of 26 days, by counting the number of empty versus full cysts on the bottom of each well. Germination was evaluated as successful if an empty cyst wall was found and unsuccessful if the cyst remained intact and did not germinate after the incubation period. Germination success was calculated as number of germinated cysts relative to the total number of cysts per well and plotted over time.

### 2.4 Selection at germination level

**Figure 2:**
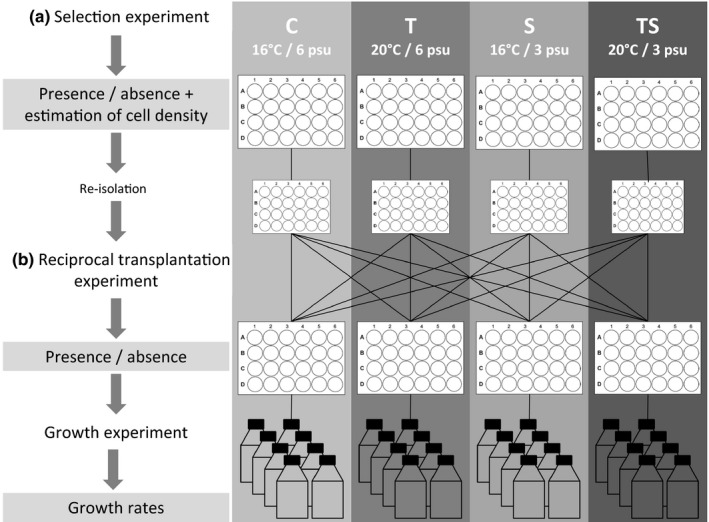
Setup of the main experimental series: (a) Germination experiment to define the life stage susceptible to selection, (b) reciprocal transplantation and growth experiments to assess if *A. ostenfeldii* is adapted to specific germination conditions. Capital white letters indicate the treatment

The purpose of our selection experiment (Figure 2a) was to find out if the final germination success is affected by different temperature and salinity scenarios and to identify where selection happens: during germination or later in the vegetative stage of the life cycle (defined by the amount of cells established per cyst). Here, the same treatments were used as for the first germination experiment, but this time 96 cysts were isolated per treatment and placed individually in wells of 24‐well plates, each well containing 2 ml of f/2‐Si medium. Two weeks after start of the experiment, germination success of each cyst was recorded microscopically (Zeiss Stemi SV 11 stereo microscope and Leica DMI3000 B inverted microscope). Germination was evaluated as successful if at least one empty cyst and vegetative cells or several immobile cells were present and unsuccessful if one full cyst was present. Additionally, the number of vegetative cells (originating from one cyst) was estimated for each well to assess how vegetative growth is affected immediately after germination and grouped in categories: <10, 11–50, 51–100, and >100 cells. Four weeks after the start of the experiment, cysts, which were not germinated yet, were re‐inspected and the germination success recorded. The response of the strains within these first four weeks after germination is unaffected by any further processing steps, like re‐isolation of single cells and transfer of cultures to larger containers, which can affect survival of some strains, and therefore considered relevant.

### 2.5 Adaptation to germination conditions

Clonal cultures established from successfully germinated resting cysts of the selection experiment were used to perform reciprocal transplantation experiments (Figure 2b). The aim of these experiments was to test whether strains which had germinated under specific conditions of the selection experiment (C, S, T, and TS) were adapted to those conditions. If the strains were adapted to their germination conditions, one would expect them to grow best at the same conditions. Transplantation to other conditions, in contrast, would result in lower growth rates. Higher growth rates after transplantation would indicate that strains are not adapted to their germination conditions, but they can acclimate to different conditions rapidly and proof at the same time that selection does not happen during germination. For this purpose, eight well‐growing cultures of each treatment of the germination experiment were randomly selected. Single cells were re‐isolated after 2 weeks (after 4–5 divisions) to establish clonal cultures, followed by a short culturing period of 8 weeks (around 10 divisions) under the same conditions, to increase the cell number for the transplantation experiment. The culturing period was kept as short as possible to minimize a potential acclimation to the respective growth conditions. After the short culturing period, single vegetative cells were transplanted reciprocally from respective germination conditions to all other conditions. Strains which were transplanted to the same growth conditions as their initial germination conditions (from C to C, S to S, T to T or TS to TS) served as controls in this experiment. For phenotypic characterization of the transplanted strains, growth experiments were carried out in tissue culture flasks, with a starting concentration of 500 cells per ml and a total volume of 40 ml. Growth was inferred from the development of Chl *a* fluorescence in each flask. Samples were measured at the start of the experiment, 7 days later and every 3–4 days for a period of one month after that with a fluorescence spectrophotometer (Varian Cary Eclipse; excitation 440 nm, emission 680 nm) equipped with a well plate reader. Growth rates (*r*, rate of increase) were calculated based on the longest period of exponential growth using the equation *r* = ln (*N_t_*/*N*_0_)/Δ*t* (Wood et al., 2005). The interval of exponential growth was determined from growth curves established for each experimental culture and included at least three time points.

### 2.6 Statistical analysis

All statistical analyses were performed with R version 3.4.4 (R Core Team, 2018) and RStudio (RStudio Team, 2015). For the selection experiment, a Pearson's Chi‐squared test was performed to check if the treatment has an effect on the ratio of germinated versus not germinated cysts. A simple linear regression was applied to model the estimated abundance of vegetative cells after germination as function of the treatment, followed by an analysis of variance (ANOVA). The variate “treatment” was categorical with four levels (C, S, T, and TS).

For the statistical analysis of the transplant experiment data, we distinguished between two effects: (1) if the growth conditions after transplantation were the same as before (coded as 0) or different (coded as (1) and (2) if conditions after transplantation were different from the germination condition, which other treatment (C, S, T, or TS) was applied. Same conditions before and after transplantation served as controls (e.g., transfer from C to C, S to S, T to T or ST to ST). A linear regression was used to model the growth rate after transplantation, followed by a simple two‐way ANOVA with two categorical predictors (2 levels and 4 levels) and the interaction between them. The strain could not be included as a random effect, because our experimental setup only included one observation per strain for each treatment combination, which made it impossible to separate a treatment effect from the strain effect. Model assumptions were verified by plotting residuals versus fitted values.

## 3 RESULTS

### 3.1 Germination success

**Figure 3:**
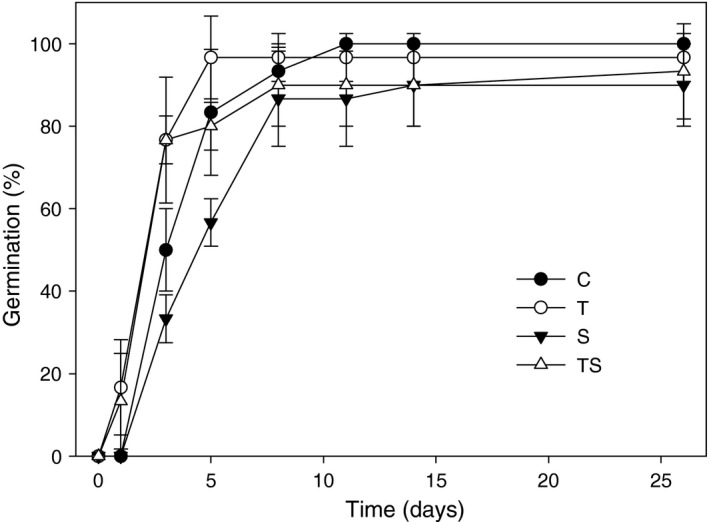
Cumulative percentage of *A. ostenfeldii* resting cysts, germinated under different experimental conditions: 16°C, 6 psu (C); 20°C, 6 psu (T); 16°C, 3 psu (S); 20°C, 3 psu (TS). Three replicates per treatment with 10 cysts in each (means ± standard deviation, *n* = 3)

During the first germination experiment, cysts of *A. ostenfeldii* germinated rapidly after exposure to favorable conditions (Figure 3). Regardless of the treatment, more than 75% of the cysts germinated within 8 days. The fastest germination was observed in the T and TS treatment, where cells occurred already one day after setting up the experiment, and lower salinity (S) resulted in slightly delayed germination (starting at day 3). The final germination success ranged from 80%–100% after 25 days.

### 3.2 Selection at germination level

**Figure 4:**
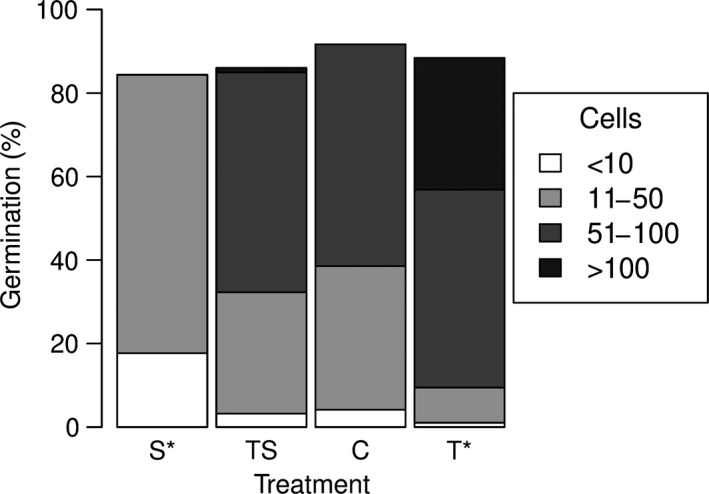
Percentage of *A. ostenfeldii *resting cysts germinated, relative to the total amount of cysts isolated for each treatment, two weeks after incubation. Control (C): 16°C, 6 psu, *n* = 94; high temperature (T): 20°C, 6 psu, *n* = 95; low salinity (S): 16°C, 3 psu, *n* = 96; high temperature combined with low salinity (TS): 20°C, 3 psu, *n* = 93. Colors of the stacked bars represent the estimated abundances of vegetative cells originating from each cyst grouped in categories: <10 cells, 11–50 cells, 51–100 cells, and >100 cells. Asterisks indicate treatments significantly different from the control with respect to the cell numbers (*p* < 0.001)

In our selection experiment, more than 84% of the cysts had germinated after two weeks of incubation, regardless of the treatment (Figure 4). The highest germination success was observed for the control, reaching 92%, followed by treatment T, TS, and S with 88%, 86%, and 84%, respectively. We found no significant difference between the ratio of germinated and not germinated cysts between the treatments (*χ* ^2^ = 3.27, *df* = 3, *p* = 0.35). When modeling the estimated abundance of vegetative cells after germination as function of the treatment, a significant effect of the treatment was found (ANOVA: *F*_3,328_ = 86.74, *p* < 0.001) with an *R* ^2^ of 0.44. Estimation of the cell numbers established per resting cyst revealed that high temperature (*T*) supported significantly higher cell numbers, compared to the control, (*t* = −8.07; *df* = 328, *p* < 0.001) whereas low salinity (S) resulted in significantly lower cell numbers, compared to the control (*t* = −8.31; *df* = 328, *p* < 0.001) (Figure 4).

### 3.3 Adaptation to germination conditions

**Figure 5:**
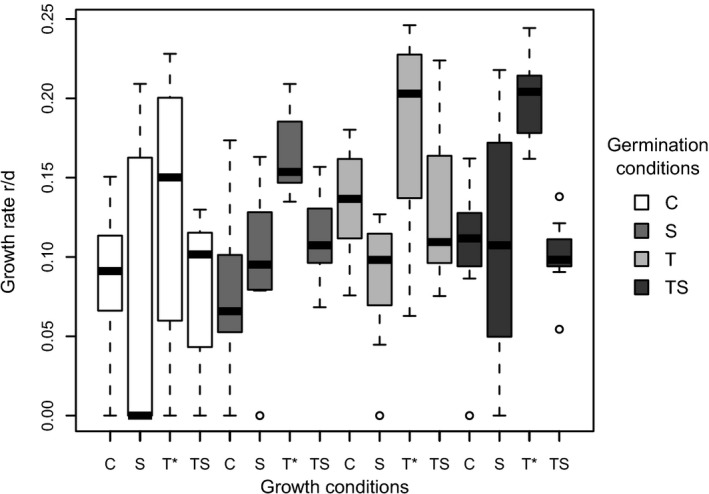
Box‐and‐whisker plots showing growth rates of strains transplanted from germination conditions (indicated by the color of the bars) reciprocally to the growth conditions. Control (C): 16°C, 6 psu; high temperature (T): 20°C, 6 psu; low salinity (S): 16°C, 3 psu; high temperature combined with low salinity (TS): 20°C, 3 psu; *n* = 8 for all treatments (except for T and TS germinated under condition TS, *n* = 7). Asterisks indicate treatments significantly different from the control (*p* < 0.05). The plots include the lower and upper extremes (horizontal lines outside the box), 25th and 75th percentiles (top and bottom frame of the box) and the median (solid line within each box)

To test if germinated *A. ostenfeldii* strains from the selection experiment (Figures 2a and 4) are adapted to their germination conditions, a subset of these strains were used to perform a reciprocal transplantation experiment (Figures 2b and 5). Overall, growth rates were affected by growth rather than germination conditions. Transplantation to higher temperature resulted in higher growth rates for most strains, and also the maximal growth rates were observed after transplantation to T, at all germination conditions. When modeling the growth rate as function of the germination condition in combination with the growth condition, a significant effect of the growth condition was found (two‐way ANOVA, *F*_7,118_ = 11.87, *p* < 0.001). Additionally, growth rates of strains growing at condition T were significantly different from the control (*t* = 3.28; *df* = 118, *p* < 0.01). The germination condition, as well as the interaction between different germination and growth condition, had no significant effect on the growth rates. Strains with the same germination and growth conditions (e.g., transplantation from C to C) served as a control in this experiment and the control of treatment C (16°C and 6 psu) had an average growth rate of 0.09/day. After transfer from C to S, high response variability was observed. The majority (62.5%) of the strains did not grow, and among the growing strains, 25% exceeded the maximum growth rate of the control. Similarly, transplantation from C to T resulted in highly variable growth rates, but with higher mean growth rate (0.13/day). Transplantation from C to TS did not alter growth rates substantially but resulted in a similar mean growth rate as the reference (0.08/day). The growth rates of the reference S (mean 0.10/day) were comparable to C, but showed less variation. When transplanted from S to C the mean growth rate dropped to 0.08/day and greater variation occurred. After transplantation from S to T and TS, growth rates increased on average to 0.16 and 0.11/day, respectively. For strains germinated at condition T, different responses to transplantation were observed. We measured the highest growth rates (mean 0.18/day) for the control and transplantation to all other treatments led to reduced growth. Different responses to transplantation were also recorded for strains germinated at condition TS. Transfer from TS to C did not alter the growth rates substantially (mean 0.10/day). Transplantation from TS to S resulted in a highly variable response, which is comparable to the transplantation from C to S and TS to T caused the highest mean growth rates of all treatments (0.20/day).

## 4 DISCUSSION

We were aiming to define if certain strains from the diverse *A. ostenfeldii* cyst pool are selected by future temperature and salinity conditions and if selection acts at the level of cyst germination or later in the active phase of the life cycle. Additionally, we investigated if strains which germinated at a specific temperature or salinity are adapted to those conditions or if their phenotypic response depends on the conditions after germination. We hypothesized that (a) temperature and salinity affect the final germination success of *A. ostenfeldii* cysts and (b) successfully germinated strains are adapted to respective temperature and salinity conditions. Contrary to our hypothesis, the majority of *A. ostenfeldii* resting cysts, isolated randomly from the seed bank of Baltic surface sediments, are able to germinate within a short time after exposure to all tested conditions. Both factors, however, affected growth after germination significantly. The higher relevance of conditions for growth compared to germination was emphasized by the results of the transplant experiments where growth rates of reciprocally transplanted strains were a response to the growth conditions after transplantation, rather than their germination conditions. Higher temperature caused significantly higher growth rates, and in addition, we observed strong variation in phenotypic responses (growth) after transplantation.

### 4.1 Selection at germination level

In contrast to our expectation, there was no significant influence of the tested salinities and temperatures on germination rate, which was over 75% at all conditions. Changes of these environmental factors in the tested ranges thus do not seem to act as filters, preventing germination of unsuited strains. Slightly delayed germination at lower salinity and quicker germination at high temperature suggest that there is a weak effect of both environmental factors on germination (Figure 3). It led to a time difference of approximately five days between the maximum numbers of cysts germinated at respective treatments. This time span is very short in relation to the long growth season of *A. ostenfeldii* (Hakanen et al., 2012); therefore, we do not consider these effects relevant for the composition of the populations initiating bloom formation in nature. Our results are comparable to other studies on germination behavior of dinoflagellates, showing that environmental factors can slow down or accelerate germination, without affecting the final germination success (Binder & Anderson, 1987; Blanco et al., 2009; Genovesi et al., 2009; Moore et al., 2015). Similar germination behavior is well documented for plants, where suboptimal temperature can lead to a slower germination rate, but still allow successful germination (Bewley & Black, 1982). In contrast, environmental factors can represent strong germination filters, allowing for natural selection in plants (Donohue et al., 2010). Natural selection on germination timing of *Arabidopsis thaliana* was, for example, suggested to be an efficient sieve that can determine which genotypes can persist in different geographic locations (Donohue et al., 2005). The relevance of temperature as environmental filter for germination was recognized for several cold‐water dinoflagellates (Kremp & Anderson, 2000; Rengefors & Anderson, 1998), but temperature and salinity limits for germination of Baltic *A. ostenfeldii* have not been defined yet. We know that at least temperature becomes selective at extreme values (Jerney et al., unpublished data), which might be also true for salinity, but not in the relatively narrow range tested in our study. If germination of *A. ostenfeldii *resting stages remains unaffected by the tested temperature and salinity conditions, a large fraction of the seed bank could potentially germinate as soon as conditions become suitable. In unpredictable environments, the prevention of germination of some seeds through dormancy was suggested to reduce the risk of extinction if conditions were to turn unfavorable after germination (Donohue et al., 2010). We assume that Baltic *A. ostenfeldii* populations do not exhibit a strongly pronounced dormancy period, since cysts are able to germinate throughout the year at a high rate (Jerney et al., unpublished data). A more realistic scenario is that under suitable conditions indeed a large part of the population germinates and is able to tolerate a broad range of environmental conditions thereafter. In addition, vegetative cells may form temporary resting cysts to escape unsuitable conditions again, as frequently observed in cultures.

### 4.2 Postgermination selection

In contrast to our first hypothesis, the environmental factors tested, herein, did not select for certain strains during germination, but became relevant during the growth phase, shortly after germination. The observed effects of temperature and salinity on growth of *A. ostenfeldii* in our study are comparable to earlier findings (Kremp et al., 2012, 2009; Suikkanen et al., 2013) and underpin our assumption that temperature and salinity exert selection pressure on the growing population. The direct response to selection depends on the level of standing genetic variation in a population (Bell & Collins, 2008) and by supporting or suppressing growth of certain strains temperature and salinity have the potential to affect the genetic composition of future populations. Baltic *A. ostenfeldii* populations seem to be geno‐ and phenotypically diverse (Kremp et al., 2016), suggesting that suitable individuals can be selected under environmental change.

We found that reduced salinity was unfavorable and higher temperature beneficial for many strains, but in our TS treatment, the positive effect of higher temperature was apparently compensated by lower salinity, resulting in growth rates similar to the control. The two selected, climate change‐related parameters have apparently opposite effects on growth, which highlights the importance of studying the effect of multiple environmental factors simultaneously, since they may result in a different response, compared to single factors. Although the TS treatment, as the most likely for future conditions, did not result in the highest growth rates, in most cases it was very comparable to the control (present conditions), indicating that *A. ostenfeldii* seems to be “well‐prepared” for the future. As shown by our experiments, suitable strains exist and there should be enough genetic variance in the population (Kremp et al., 2016) to support adaptation.

### 4.3 Acclimation and trait variability

Our second hypothesis was that successfully germinated strains are adapted to respective temperature and salinity conditions and will grow best at those conditions. In contrast to our expectations, successfully germinated strains were not adapted to germination conditions. Instead, they were able to adjust to temperature and salinity different from their germination conditions and even outperform nontransplanted control strains. This indicates that *A. ostenfeldii* has a broad germination and growth tolerance for the tested range of temperature and salinity and a high acclimation potential, which is common for phytoplankton living in fluctuating ecosystems (Collins et al., 2013). Offspring will most likely encounter conditions different from its parents, but will adjust to them by phenotypic plasticity (Bell & Collins, 2008). The lack of adaptation to temperature and salinity conditions found in our study seems to contradict earlier observations, which proposed that different responses to temperature and salinity changes reflect adaptations to respective conditions (Kremp et al., 2016).

Moreover, we observed high growth rate variability after transplantation, when comparing growth rates within each set of transplanted strains, showing that responses to temperature and salinity changes can be strain specific. High strain‐specific trait—or phenotypic—variability has been demonstrated for this species before (Kremp et al., 2016; Suikkanen et al., 2013) and underlines how crucial it is to test responses to environmental conditions with a large number of strains. We used several strains for our experiments, and effects of our treatments have probably been masked by high‐trait variability. High intraspecific trait variation has recently been linked to fluctuating selection pressures (Brandenburg et al., 2018) and might partly explain this species’ success in shallow waters of the Baltic Sea. The high‐trait variability makes it difficult to assess the overall effect of environmental factors on the population, but having a high variability of responses seems be part of the generalist life strategy of this species and the additive genetic variance may often be sufficient to support adaptation to rapid environmental change (Bell & Collins, 2008).

## 5 CONCLUSIONS

Germination success of *A. ostenfeldii* resting stages remained unaffected by temperature and salinity, indicating that these factors, in the tested range, do not filter suitable individuals through prevention of germination. However, both parameters exert selection pressure on the vegetative population and thus have the capability to shape the phenotype composition of populations. It is likely that *A. ostenfeldii* populations can persist under predicted future climate conditions (warmer Baltic Sea with lower salinity), due to its flexible germination behavior, high plasticity, and a great adaptation potential, based on large geno‐ and phenotypic diversity.

## CONFLICT OF INTEREST

None declared.

## AUTHOR CONTRIBUTIONS

JJ, AK, and SS conceived the study. JJ collected the samples. JJ, AK, SS, and EL carried out experimental work and supported data collection. JJ carried out data analysis. JJ and AK lead the writing of the manuscript with input from all authors.

## Data Availability

Germination and growth rate data can be accessed at Dryad.
